# The role of dopamine release and D2 dopamine receptor in GHRH and somatostatin cells in controlling growth hormone secretion

**DOI:** 10.3389/fendo.2025.1741139

**Published:** 2026-01-12

**Authors:** Gabriel O. de Souza, Daniela O. Gusmao, Maria E. de Sousa, Marina G. Martins, Alexandre S. Basso, Jose Donato

**Affiliations:** 1 Departmento de Fisiologia e Biofísica, Instituto de Ciencias Biomedicas, Universidade de Sao Paulo, Sao Paulo, Brazil; 2 Departamento de Microbiologia, Imunologia e Parasitologia, Escola Paulista de Medicina, Universidade Federal de Sao Paulo, Sao Paulo, Brazil

**Keywords:** dopamine, GH, hypothalamus, IGF-1, neuroendocrinology

## Abstract

**Introduction:**

Pituitary growth hormone (GH) secretion is primarily controlled by GH-releasing hormone (GHRH) and somatostatin (SST), peptides produced by hypothalamic neurons. Evidence indicates that dopamine also modulates GH secretion, potentially via D2 dopamine receptor (D2R). Additionally, a subset of GHRH neurons in the arcuate nucleus of the hypothalamus expresses tyrosine hydroxylase (TH), the rate-limiting enzyme of dopamine biosynthesis. However, the role of dopamine release from GHRH neurons and the neuronal population that expresses D2R to regulate GH secretion remain currently unknown.

**Methods:**

Mice lacking TH specifically in GHRH cells were generated.

**Results:**

GHRH^ΔTH^ mice display relatively normal body growth and pulsatile GH secretion compared to control animals. Next, the effects of D2R deletion in GHRH or SST neurons were examined. GHRH^ΔDrd2^ male mice tended to have reduced lean mass and increased adiposity compared to controls, along with decreased basal GH secretion. SST^ΔDrd2^ male mice also exhibited reduced body weight and lean mass. Total and pulsatile GH secretion, as well as serum insulin-like growth factor 1 (IGF-1) levels, were not different between groups. No significant differences in body growth, GH secretion pattern, and serum IGF-1 concentration were observed among control, GHRH^ΔDrd2^, and SST^ΔDrd2^ females.

**Discussion:**

Dopamine production in GHRH neurons is not necessary for regulating body growth or GH secretion. D2R ablation in GHRH or SST neurons has a small impact on lean mass and GH secretion, indicating that these neurons mediate only a minor part of the effects induced by complete D2R absence in male mice.

## 1 Introduction

Pituitary growth hormone (GH) secretion is primarily regulated by hypothalamic neurohormones (1–3). In this regard, neurons in the hypothalamus produce GH-releasing hormone (GHRH), which stimulates GH secretion, while somatostatin (SST) inhibits the activity of somatotropic cells (1–3). These peptides are released in the median eminence by hypophysiotropic neurons and travel to the anterior pituitary via the hypophyseal portal system (1–3). Besides the well-established roles of GHRH and SST in regulating GH secretion, other neuromodulators may also directly or indirectly regulate GH production. For example, several studies have shown that neuropeptide Y influences GH secretion by affecting the activity of either GHRH or SST neurons (4–8). Ding et al. 2025 have shown that noradrenergic neurons in the locus coeruleus control GH secretion by increasing wakefulness (9).

GH secretion is regulated by negative feedback loops (1–3). Our research group has mapped the distribution of GH-responsive neurons using the ability of a high-dose GH injection to induce the phosphorylation of the signal transducer and activator of transcription 5 (pSTAT5) (10, 11). Using this method, we demonstrated that tyrosine hydroxylase (TH)-expressing neurons in the arcuate nucleus of the hypothalamus (ARH) express GH-induced pSTAT5 (12, 13). ARH^TH^ neurons produce dopamine, and they classically regulate prolactin secretion (14). Notably, knocking out the GH receptor (GHR) in TH-expressing cells results in increased body growth and GH secretion in male mice, typical signs of negative feedback loss. Conversely, GHR deletion in dopamine beta-hydroxylase neurons, which are essential for converting dopamine into noradrenaline, does not impact growth or GH secretion. These findings collectively indicate that dopamine neurons are involved in the negative feedback regulation of the GH axis (13). Several studies have shown that a subset of ARH^GHRH^ neurons expresses TH (13, 15–18). Therefore, GHRH and dopamine can be coreleased by a subset of ARH neurons. However, the role of dopaminergic transmission in GHRH neurons to regulate GH secretion and, consequently, body growth has not been studied to date. Thus, the first objective of the present study was to investigate whether mice lacking TH in GHRH neurons present alterations in body growth and GH secretion.

Dopamine controls GH secretion probably via the D2 dopamine receptor (D2R). In accordance with this idea, D2R agonists, such as cabergoline, influence GH secretion and are used to treat acromegaly (19, 20). Additionally, D2R knockout mice exhibit decreased body growth and reduced pituitary GH secretion (21, 22). Of note, this effect is seen only in male mice (21). D2R expression is found in the hypothalamus and pituitary gland, so dopamine might regulate GH secretion through both tissues. However, mice with a neuron-specific D2R deletion also show reduced body growth, indicating that central dopamine signaling likely controls the GH axis (23). In the rodent hypothalamus, D2R expression is observed in the ARH, periventricular nucleus (PV), and paraventricular nucleus (PVH), regions where GHRH and SST hypophysiotropic neurons are located (24, 25). Given this information, it is plausible to hypothesize that GHRH and SST neurons may express D2R and thus be influenced by dopaminergic transmission. Therefore, our second goal was to determine whether D2R expression in GHRH- or SST-expressing neurons is necessary for regulating GH secretion and body growth in male and female mice.

## 2 Materials and methods

### 2.1 Animals

To inactivate the *Th* gene in GHRH-expressing cells, GHRH^Cre^ mice (26) (RRID: IMSR_JAX:031096, The Jackson Laboratory, Bar Harbor, ME, USA) were crossed to TH^flox/flox^ animals (27) until generating TH^flox/flox^::GHRH^Cre^ mice (named GHRH^ΔTH^) and their respective control animals (TH^flox/flox^). For histological experiments, GHRH^Cre^ mice were crossed with Rosa26^CAG-LoxPSTOPLoxP-eGFP-L10A^ mice (26), leading to the expression of enhanced green fluorescent protein (eGFP) only in Cre-expressing cells. GHRH^Cre^ and SST^Cre^ mice (RRID: IMSR_JAX:018973; The Jackson Laboratory) were crossed with Dr2d^flox/flox^ mice (RRID: IMSR_JAX:020631; The Jackson Laboratory), generating Drd2^flox/flox^::GHRH^Cre^ mice (named GHRH^ΔDrd2^), Drd2^flox/flox^::SST^Cre^ mice (named SST^ΔDrd2^), and the control group (Drd2^flox/flox^). Mice were maintained in the C57BL/6J background and housed in a 12-h light/dark cycle, with lights on at 8:00. The mutations were genotyped using polymerase chain reaction on DNA extracted from the tail tip (REDExtract-N-Amp™ Tissue PCR Kit, MilliporeSigma, St. Louis, MO, USA). The animal procedures were approved by the Ethics Committee on the Use of Animals of the Institute of Biomedical Sciences at the University of São Paulo.

### 2.2 Immunofluorescence staining

Mice expressing eGFP in GHRH neurons were perfused with saline, followed by formalin. Thirty-µm-thick brain sections were obtained using a freezing microtome. Then, brain slices were rinsed in 0.02 M potassium phosphate-buffered saline (PBS), pH 7.4 (KPBS), and incubated for 1 hour in 3% normal serum, followed by an overnight incubation in an anti-TH antibody (1:1000; Abcam, Cambridge, UK; Cat# ab112; RRID: AB_297840). Sections were rinsed in KPBS and incubated for 90 minutes with Alexa Fluor 594-conjugated secondary antibody (1:500, Jackson ImmunoResearch Laboratories, Cambridge, MA). After rinsing in KPBS, sections were mounted onto gelatin-coated slides and covered with Fluoromount G (Electron Microscopic Sciences, Hatfield, PA). Photomicrographs were obtained using an AxioImager A1 microscope (Zeiss, Munich, Germany) equipped with a Zeiss Axiocam 512 camera. The percentages of single- and double-labeled neurons were analyzed in the ARH using Adobe Photoshop.

### 2.3 RNAscope

RNA *in situ* hybridization was used to detect the colocalization between *Drd2* mRNA and *Ghrh* or *Sst* mRNA in the mouse brain. Briefly, mice were perfused, and their brains were harvested as previously described in the methods. Coronal sections were subjected to an RNAscope^®^ multiplex fluorescent V2 assay (#323110, ACDBio, Newark, CA, USA) following the manufacturer’s instructions. Brain sections were rinsed in PBS, dried at 60°C for 30 minutes, and dehydrated in ethanol. After incubation with H_2_O_2_ for 10 minutes at room temperature and Protease III for 30 minutes at 40°C, sections were incubated with *Drd2* mRNA (Mm-Drd2-C2, #406501-C2, ACDBio), *Ghrh* mRNA (Mm-Ghrh, #470991, ACDBio), or *Sst* mRNA (Mm-Sst, # 404631, ACDBio) probes for 2 hours at 40°C. *Drd2* mRNA was visualized using TSA Plus^®^ Fluorescein (1:1500, #NEL741001KT, Akoya Biosciences, Marlborough, MA, USA), while *Ghrh* or *Sst* mRNA was visualized using TSA Plus^®^ CY3 (1:1500, #NEL744001KT, Akoya Biosciences). Then, slides were counterstained with DAPI, cover-slipped with ProLong Gold^®^ antifade media (#P36930, ThermoFisher Scientific), and stored in the dark at 4°C until imaging.

### 2.4 Body composition and body growth

Body weight changes were monitored over time. Body composition was determined by time-domain nuclear magnetic resonance using the LF50 body composition analyzer (Bruker, Germany). The naso-anal length was measured at the end of the follow-up period in mice under isoflurane anesthesia.

### 2.5 Evaluation of GH secretion and insulin-like growth factor 1 (IGF-1) levels

Before assessing pulsatile GH secretion, 4-week-old mice were acclimated daily to the tail-tip blood sampling procedure for one month. Then, 36 sequential blood samples were collected from the tail tip in approximately 8-week-old mice at 10-minute intervals, beginning at 9:00 a.m. (1 hour after lights-on). Blood collection started by removing a small portion of the tail tip (1 mm) with a surgical blade. 5 μL blood samples were transferred to a tube containing 105 μL of PBS with 0.05% Tween-20. After each blood collection, fingertip pressure was gently applied to the tail tip to stop the bleeding. Mice were allowed to move freely in their home cages and had *ad libitum* access to food and water throughout the blood collection period. After collection, blood samples were immediately placed on dry ice and then stored at -80 °C. Blood GH levels were analyzed using an in-house enzyme-linked immunosorbent assay (ELISA), as previously described (13, 15, 28–33). This protocol was adapted from Steyn et al. (34). GH pulses were identified using the DynPeak pulse detection algorithm (35). The analysis of the pattern of GH secretion calculated total, pulsatile, and basal GH secretion, as previously described (28, 30). Serum IGF-1 levels were measured using a commercially available ELISA kit (#MG100; RRID: AB_2827989; R&D Systems, Minneapolis, MN, USA) from trunk blood samples.

### 2.6 Statistical analysis

The differences between any two groups were analyzed using a two-tailed unpaired Student’s t-test. When comparing three groups simultaneously, one-way ANOVA followed by the Newman-Keuls multiple comparisons test was employed. Changes over time in body weight, lean mass, and fat mass were analyzed using two-way repeated-measures ANOVA. Statistical analyses were conducted with Prism software (GraphPad, San Diego, CA). All results are presented as mean ± standard error of the mean.

## 3 Results

### 3.1 TH expression in GHRH cells is not necessary to regulate body growth

**Figure 1:**
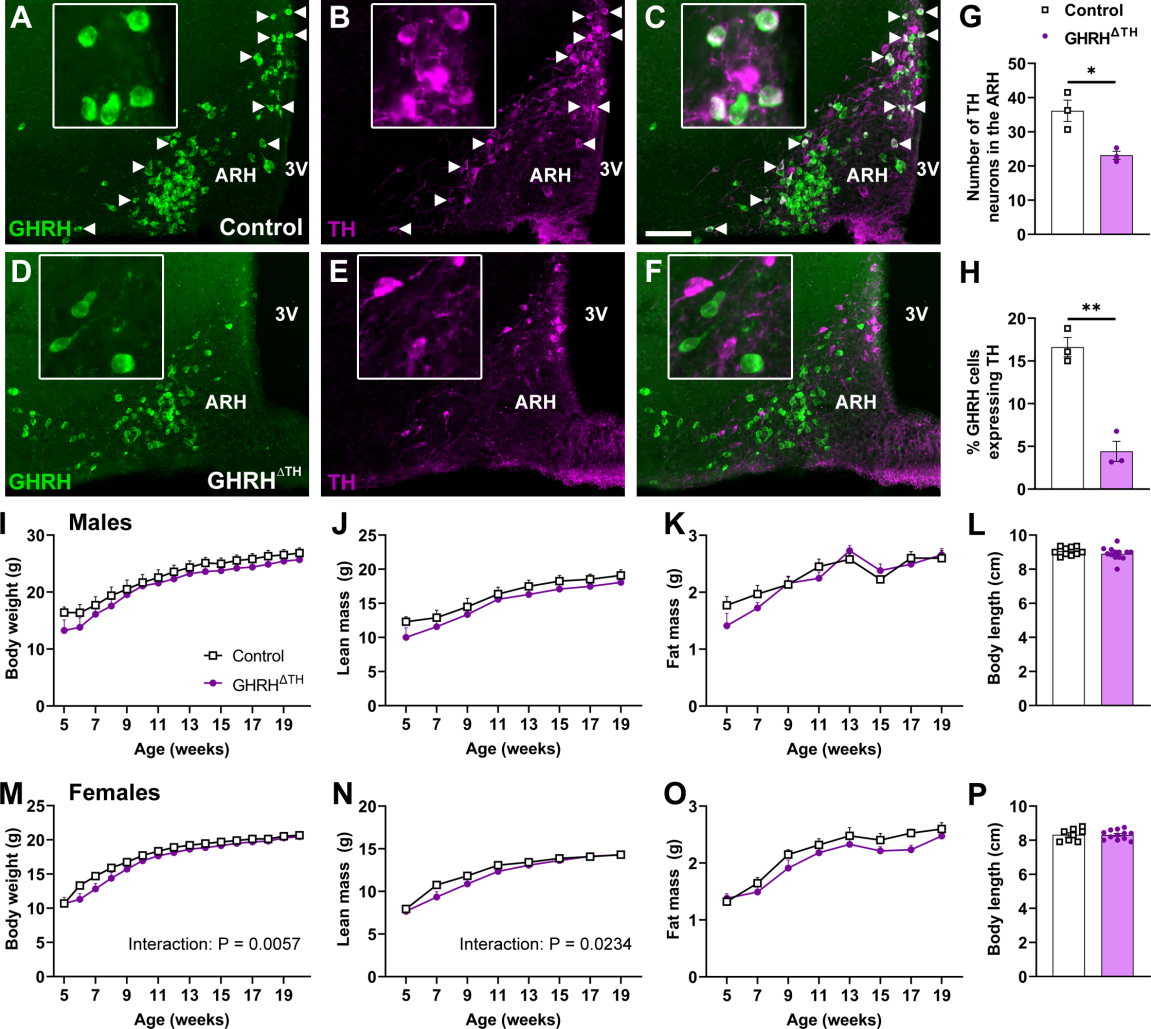
TH expression in GHRH cells is not necessary to regulate body growth. **(A–C)** Epifluorescence photomicrographs showing the colocalization between GHRH (eGFP expression) and TH (magenta) in control mice. Arrowheads indicate double-labeling cells. Scale bar = 100 µm. 3V, third ventricle; ARH, arcuate nucleus of the hypothalamus. **(D–F)** Epifluorescence photomicrographs showing the colocalization between GHRH and TH in GHRH^ΔTH^ mice. The insights are higher-magnification photomicrographs of specific areas in each figure. **(G, H)** Quantification of the number of TH-immunoreactive neurons in the ARH and the percentage of ARH^GHRH^ neurons expressing TH in control (*n* = 3) and GHRH^ΔTH^ mice (*n* = 3). *P < 0.05; **P < 0.01 (two-tailed unpaired Student’s t-test). **(I–K)** Body weight, lean mass, and fat mass over time and body length in control (*n* = 9) and GHRH^ΔTH^ (*n* = 10) male mice. **(L)** Body length in control (*n* = 10) and GHRH^ΔTH^ (*n* = 13) male mice. **(M–O)** Body weight, lean mass, and fat mass over time in control (*n* = 9) and GHRH^ΔTH^ (*n* = 12) female mice. **(P)** Body length in control (*n* = 8) and GHRH^ΔTH^ (*n* = 12) female mice. The interaction effect was calculated by two-way repeated-measures ANOVA.

Mice with a genetic deletion of the *Th* gene specifically in GHRH-expressing cells were generated. GHRH^ΔTH^ mice showed a reduction in the number of TH-immunoreactive cells in the ARH compared to controls (**Figures 1A–G**). Moreover, the percentage of ARH^GHRH^ neurons expressing TH was significantly decreased in GHRH^ΔTH^ mice (**Figure 1H**). No significant differences in body weight, lean mass, fat mass, or body length were observed between control and GHRH^ΔTH^ mice during the follow-up period, either in males or females (**Figures 1I–P**). Of note, a significant interaction between time and genotype was observed for body weight (P = 0.0057) and lean mass (P = 0.0234) in females. This was due to a tendency of GHRH^ΔTH^ mice to exhibit reductions in body weight and lean mass during the first weeks of follow-up in both sexes. However, this slight change was compensated for during development, leading to similar body growth in adult animals.

### 3.2 TH ablation in GHRH cells does not affect pulsatile GH secretion

**Figure 2:**
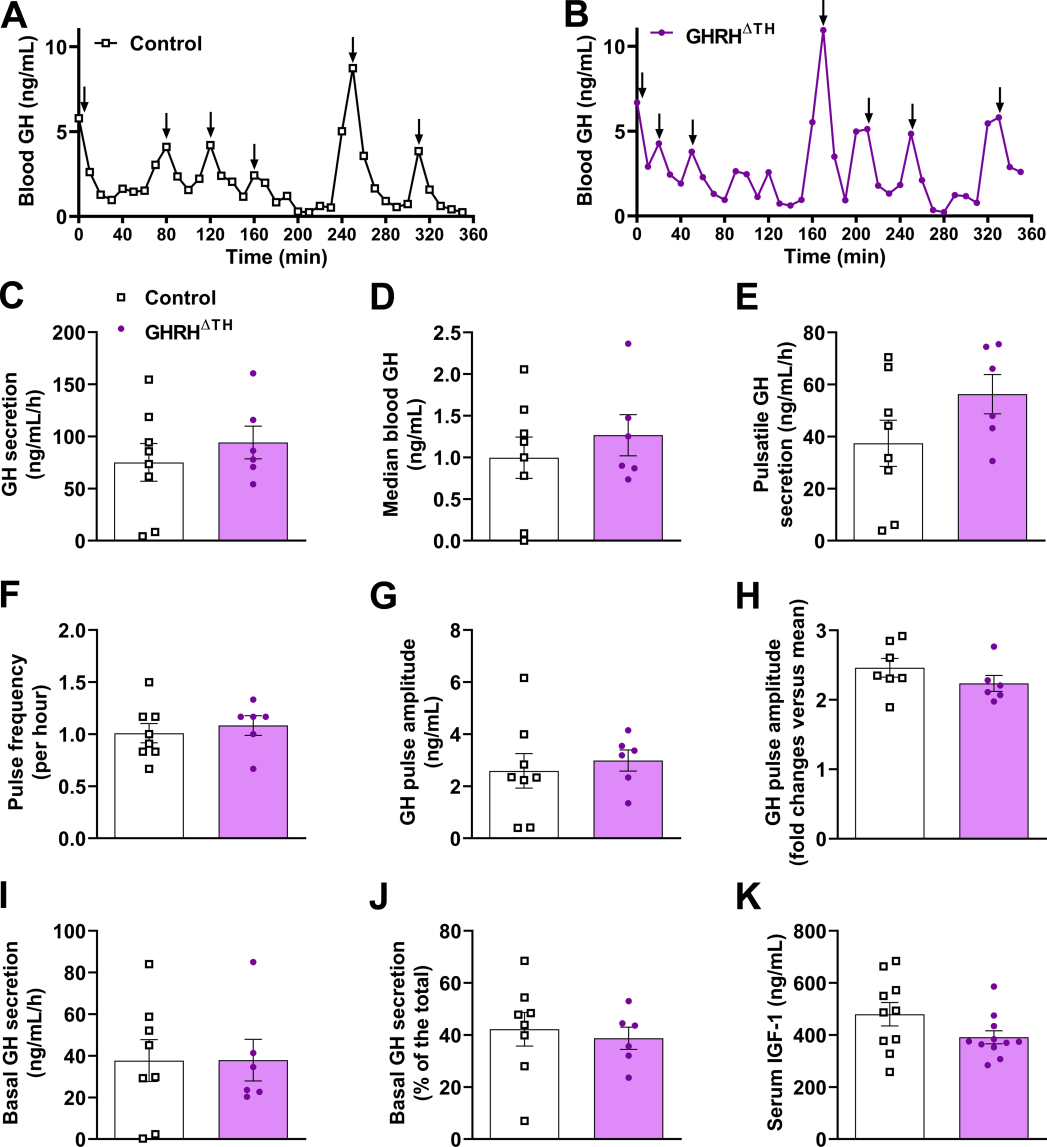
TH ablation in GHRH cells does not affect pulsatile GH secretion. **(A, B)** Representative examples of the pattern of GH secretion in a control **(A)** and GHRH^ΔTH^**(B)** male mouse. Arrows indicate GH pulses. **(C–J)** Total GH secretion, median blood GH levels, pulsatile GH secretion, GH pulse frequency, GH pulse amplitude, basal (non-pulsatile) GH secretion, and contribution of basal secretion to total GH secretion in approximately 8-week-old control (*n* = 8) and GHRH^ΔTH^ (*n* = 6) male mice. **(K)** Serum IGF-1 concentration in control (*n* = 10) and GHRH^ΔTH^ (*n* = 11) male mice.

To examine whether dopaminergic transmission in GHRH neurons affects GH secretion patterns, 36 blood samples were collected at 10-minute intervals from 8-week-old male mice (**Figures 2A, B**). GHRH^ΔTH^ male mice showed no differences in total GH secretion, median blood GH levels, pulsatile GH secretion, GH pulse frequency, GH pulse amplitude, or basal GH secretion (**Figures 2C–J**). Additionally, both control and GHRH^ΔTH^ male mice had similar serum IGF-1 levels (**Figure 2K**). In females, only serum IGF-1 levels were analyzed, with no difference between control mice (616.8 ± 50.3 ng/mL; *n* = 8) and GHRH^ΔTH^ mice (522.8 ± 53.3 ng/mL; *n* = 11; P = 0.2329).

### 3.3 Effects of D2R ablation in GHRH-expressing cells

**Figure 3:**
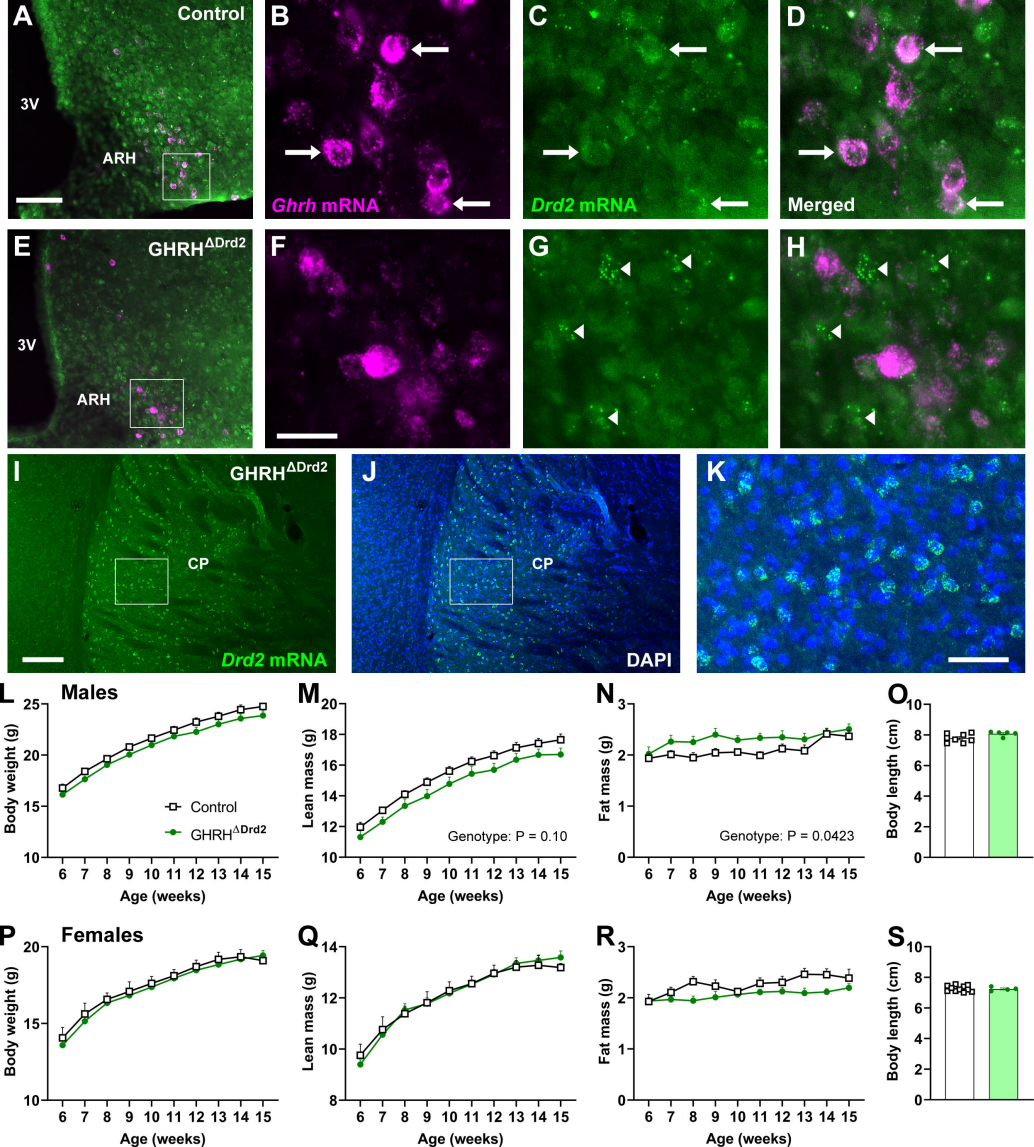
Effects of D2R ablation in GHRH-expressing cells. **(A–D)** Epifluorescence photomicrographs showing the colocalization between *Ghrh* mRNA (magenta) and *Drd2* mRNA (green) in control mice. Panels **(B–D)** show a higher magnification of the insight shown in Panel **(A)**. Arrows indicate double-labeling cells. 3V, third ventricle; ARH, arcuate nucleus of the hypothalamus. **(E–H)** Epifluorescence photomicrographs showing the lack of colocalization between *Ghrh* mRNA and *Drd2* mRNA in GHRH^ΔDrd2^ mice. Panels **(F–H)** show a higher magnification of the insight shown in Panel **(E)**. Arrowheads indicate cells that express *Drd2* mRNA but are negative for *Ghrh* mRNA. **(I–K)** Epifluorescence photomicrographs showing the expression of *Drd2* mRNA in the caudate-putamen of GHRH^ΔDrd2^ mice. Panel **(K)** shows a higher magnification of the insight shown in Panel **(J)**. Scale bars: **(A, E)** = 100 µm; **(B–D, F–H)** = 25 µm, **(I–J)** = 200 µm, K = 50 µm. **(L–N)** Body weight, lean mass, and fat mass over time in control (*n* = 11) and GHRH^ΔDrd2^ (*n* = 8) male mice. **(O)** Body length in control (*n* = 8) and GHRH^ΔDrd2^ (*n* = 5) male mice. **(P–R)** Body weight, lean mass, and fat mass over time in control (*n* = 6) and GHRH^ΔDrd2^ (*n* = 12) female mice. **(S)** Body length in control (*n* = 11) and GHRH^ΔDrd2^ (*n* = 4) female mice. The genotype effect was calculated by two-way repeated-measures ANOVA.

As mentioned earlier, male mice lacking the *Drd2* gene exhibit stunted growth, whereas females show similar overall body growth (21). This phenotype is linked to reduced pituitary GH content and GH secretory activity (22). D2R likely regulates GH secretion and body growth through a central mechanism, as neuron-specific D2R ablation also impairs body growth (23). Here, we investigated whether D2R expression in GHRH or SST neurons affects body growth and GH secretion patterns. D2R expression was found in ARH^GHRH^ neurons (**Figures 3A–D**). In contrast, GHRH^ΔDrd2^ mice showed no D2R expression in ARH^GHRH^ neurons (**Figures 3E–H**), while D2R expression remained intact in other brain regions, such as the caudate-putamen (**Figures 3I–K**). GHRH^ΔDrd2^ male mice showed a tendency to have lower body weight and lean mass (P = 0.10) compared to control animals (**Figures 3L, M**). In contrast, these mice exhibited increased fat mass (P = 0.0423) relative to controls (**Figure 3N**). By the end of the follow-up period, there were no significant differences in body length between the groups (**Figure 3O**). For females, no differences were observed in body weight, lean mass, fat mass, or body length between groups (**Figures 3P–S**).

### 3.4 D2R ablation in SST cells slightly reduces body weight and lean mass in male mice

**Figure 4:**
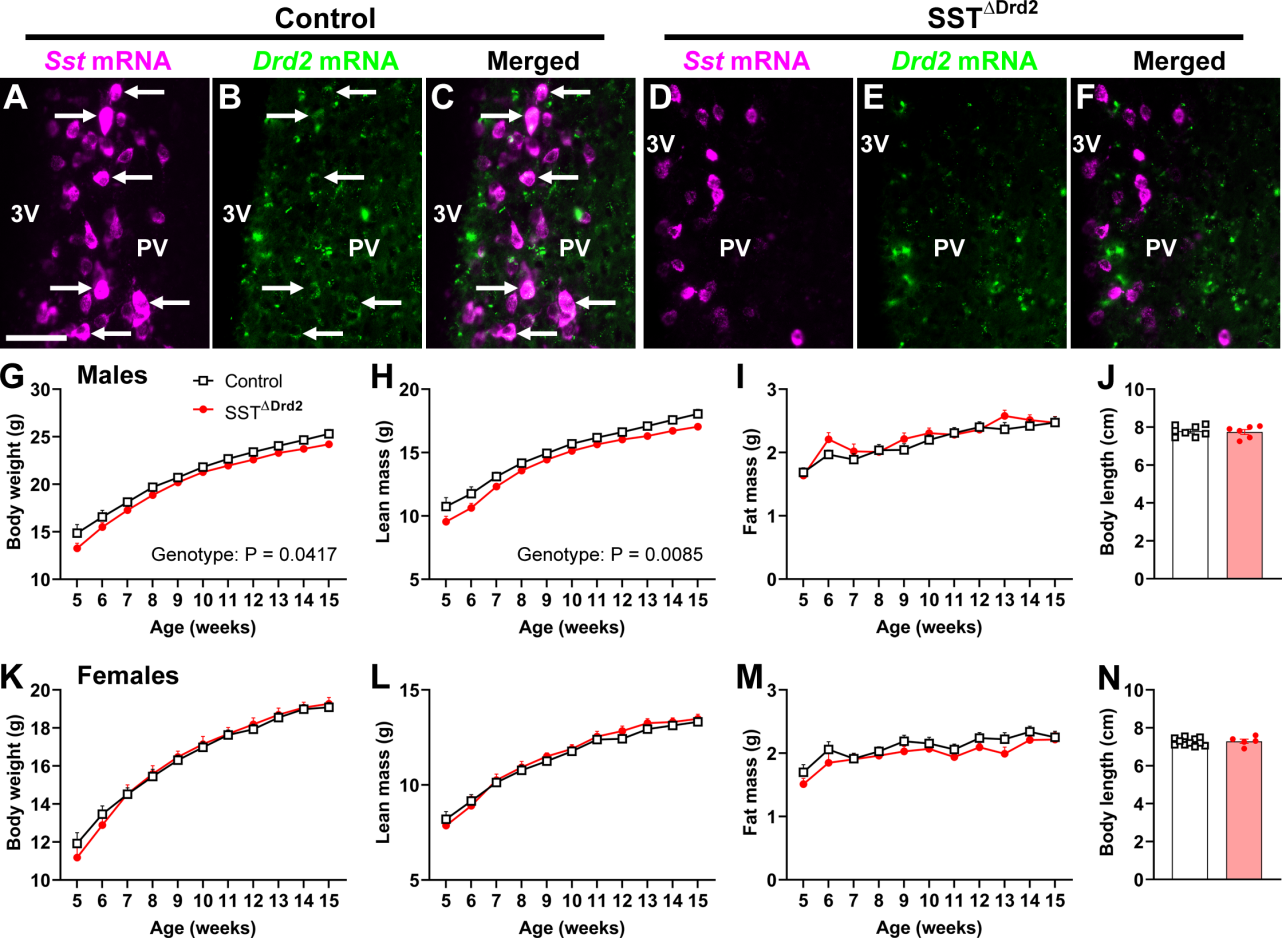
D2R ablation in SST cells slightly reduces body weight and lean mass in male mice. **(A–C)** Epifluorescence photomicrographs showing the colocalization between *Sst* mRNA (magenta) and *Drd2* mRNA (green) in control mice. Arrows indicate double-labeling cells. Scale bar = 50 µm. 3V, third ventricle; PV, periventricular nucleus of the hypothalamus. **(D–F)** Epifluorescence photomicrographs showing the lack of colocalization between *Sst* mRNA and *Drd2* mRNA in SST^ΔDrd2^ mice. **(G–I)** Body weight, lean mass, and fat mass over time in control (*n* = 11) and SST^ΔDrd2^ (*n* = 15) male mice. **(J)** Body length in control (*n* = 8) and SST^ΔDrd2^ (*n* = 6) male mice. **(K–M)** Body weight, lean mass, and fat mass over time in control (*n* = 11) and SST^ΔDrd2^ (*n* = 12) female mice. **(N)** Body length in control (*n* = 11) and SST^ΔDrd2^ (*n* = 5) female mice. The genotype effect was calculated by two-way repeated-measures ANOVA.

SST neurons in the periventricular nucleus of the hypothalamus (PV) project to the median eminence and regulate GH secretion (1, 36, 37). A subset of PV^SST^ neurons expresses D2R (**Figures 4A–C**). Mice lacking D2R in SST-expressing cells were generated (**Figures 4D–F**). In males, a slight but statistically significant reduction in body weight (P = 0.0417) and lean mass (P = 0.0085) was observed in SST^ΔDrd2^ mice compared to controls (**Figures 4G, H**). Fat mass and body length did not differ between groups (**Figures 4I, J**). In contrast, there were no differences in body weight, lean mass, fat mass, or body length between females (**Figures 4K–N**).

### 3.5 GHRH^ΔDrd2^ male mice exhibit reduced basal GH secretion

**Figure 5:**
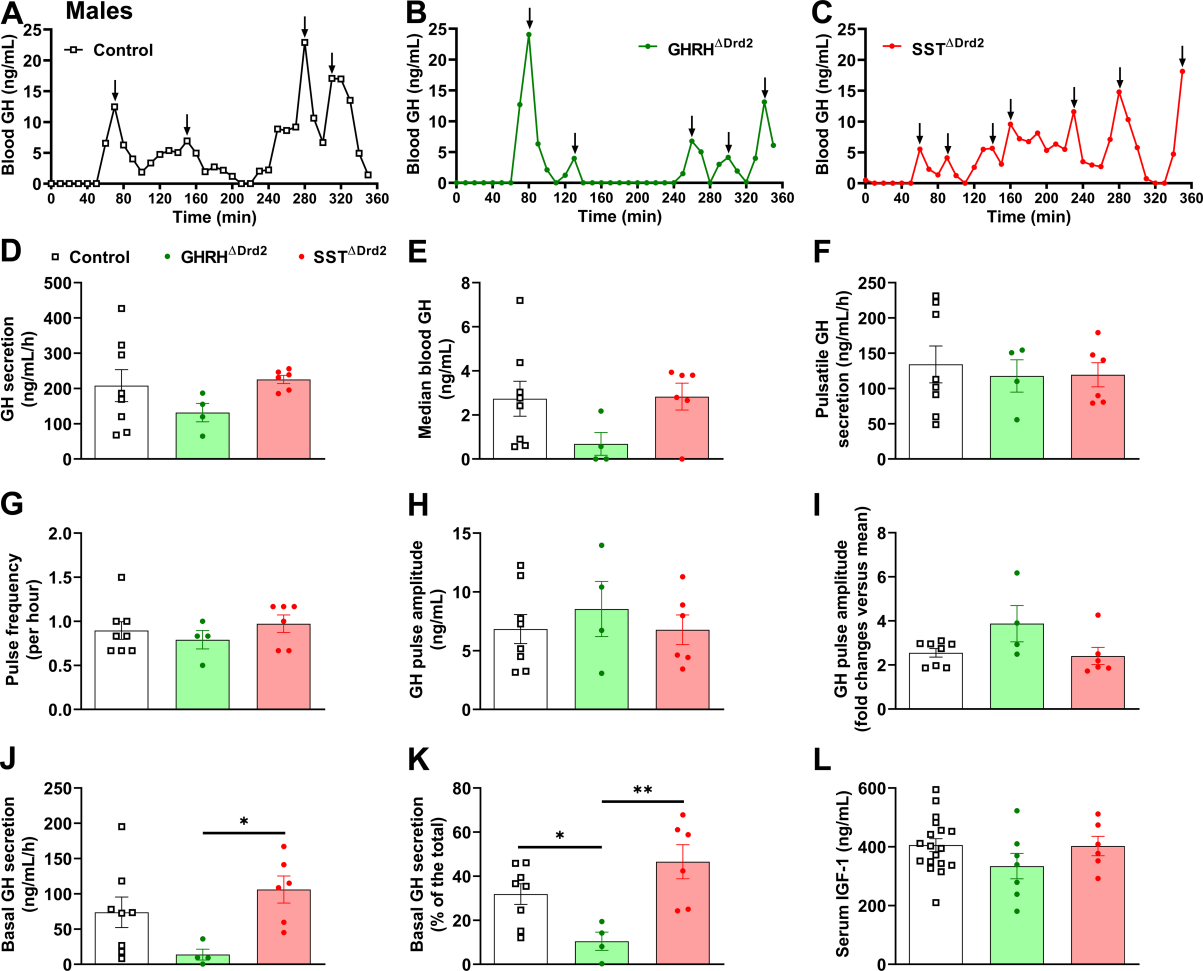
GHRH^ΔDrd2^ male mice exhibit reduced basal GH secretion. **(A–C)** Representative examples of the pattern of GH secretion in a control **(A)**, GHRH^ΔDrd2^**(B)**, and SST^ΔDrd2^**(C)** male mouse. Arrows indicate GH pulses. **(D–K)** Total GH secretion, median blood GH levels, pulsatile GH secretion, GH pulse frequency, GH pulse amplitude, basal (non-pulsatile) GH secretion, and contribution of basal secretion to total GH secretion in approximately 8-week-old control (*n* = 8), GHRH^ΔDrd2^ (*n* = 4), and SST^ΔDrd2^ (*n* = 6) male mice. **(L)** Serum IGF-1 concentration in control (*n* = 18), GHRH^ΔDrd2^ (*n* = 7), and SST^ΔDrd2^ (*n* = 6) male mice. *P < 0.05; **P < 0.01 (one-way ANOVA followed by the Newman-Keuls multiple comparisons test).

**Figure 6:**
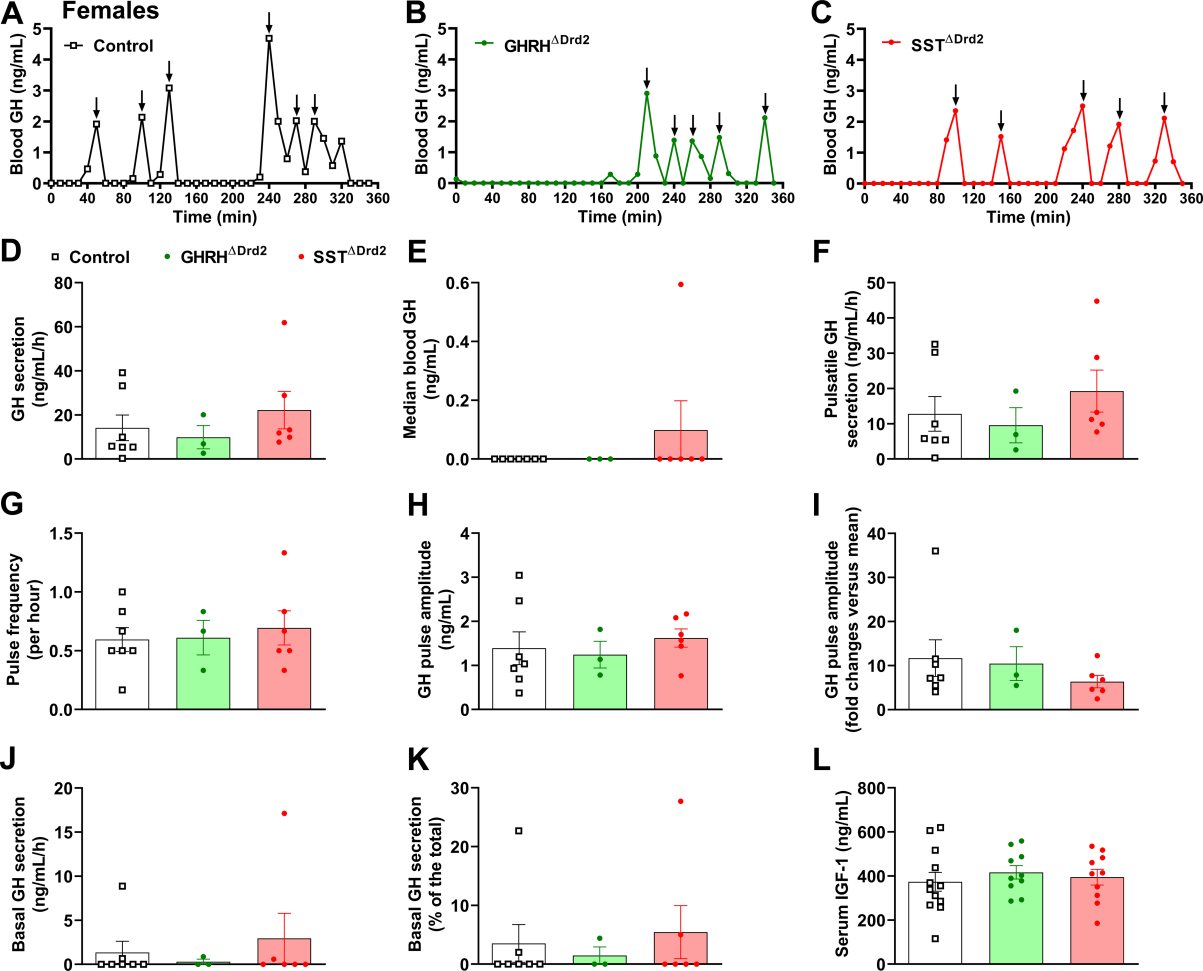
D2R ablation in GHRH or SST neurons does not alter the GH secretion pattern in female mice. **(A–C)** Representative examples of the pattern of GH secretion in a control **(A)**, GHRH^ΔDrd2^**(B)**, and SST^ΔDrd2^**(C)** female mouse. Arrows indicate GH pulses. **(D–K)** Total GH secretion, median blood GH levels, pulsatile GH secretion, GH pulse frequency, GH pulse amplitude, basal (non-pulsatile) GH secretion, and contribution of basal secretion to total GH secretion in approximately 8-week-old control (*n* = 7), GHRH^ΔDrd2^ (*n* = 3), and SST^ΔDrd2^ (*n* = 6) female mice. **(L)** Serum IGF-1 concentration in control (*n* = 12), GHRH^ΔDrd2^ (*n* = 10), and SST^ΔDrd2^ (*n* = 10) female mice.

The GH secretion patterns were analyzed in GHRH^ΔDrd2^ and SST^ΔDrd2^ mice. In males, no differences between groups were observed in total GH secretion, median blood GH, pulsatile GH secretion, GH pulse frequency, or GH pulse amplitude (**Figures 5A–I**). Interestingly, GHRH^ΔDrd2^ male mice showed reduced basal GH secretion (**Figures 5J, K**). Serum IGF-1 levels were unaffected by the mutations (**Figure 5L**). In females, total, pulsatile, and basal GH secretion, as well as serum IGF-1 levels, were unaffected by D2R ablation in either GHRH or SST neurons (**Figures 6A–L**).

## 4 Discussion

The current study tested the hypothesis that dopamine release from GHRH neurons might regulate GH secretion and, consequently, body growth. Additionally, we evaluated whether D2R expression in key neurons that control GH secretion GHRH and SST expressing cells may modulate the GH axis.

GHRH-specific TH ablation was sufficient to decrease the number of ARH^TH^ neurons significantly. It is essential to mention that some TH-positive neurons in the medial zona incerta (ZI) also express GHRH (16), so these cells were affected by genetic manipulations. These cells are classically known as the A13 dopamine group and are non-hypophysiotropic neurons (38). Additionally, anterograde tracer studies showed that cells in the medial ZI, including the A13 dopamine group, project to brain areas involved in behavior regulation, sensory input, and motor output, rather than to nuclei that contain neurons controlling the somatotropic axis, such as the ARH and PV (38, 39). Accordingly, the A13 dopamine cell group in the ZI plays a key role in nociceptive processing (40). However, the involvement of ZI^GHRH^ neurons in regulating GH secretion remains uncertain, and further studies are needed to explore this possibility.

Despite TH deletion, GHRH^ΔTH^ mice showed relatively normal body growth. A very modest reduction in body weight and lean mass was seen in the first weeks of follow-up, especially in females. However, as adults, GHRH^ΔTH^ mice had normal growth and GH secretion. Therefore, TH expression in GHRH neurons is not necessary for maintaining normal GH axis function. At most, dopaminergic transmission in GHRH neurons might influence growth rate between adolescence and adulthood, but other control systems likely compensate for the lack of TH. Since we started monitoring body weight from the fifth week of life, we have no information on whether there were differences in animal growth before then, which could indicate an impact on development during the early postnatal period. The lack of growth changes in adulthood is not necessarily unexpected, as GH secretion is controlled by numerous redundant mechanisms that involve negative feedback loops in the pituitary gland and in several hypothalamic neuronal populations (1–3, 13, 15, 18, 33). The regulation of GH secretion involves not only the negative feedback exerted by GH and IGF-1 (1, 28), but also the key roles of other hormones that control GH secretion, including ghrelin and the liver-expressed antimicrobial peptide 2 (41–43). This high redundancy in GH control is illustrated by a study that inactivated the GHR or the IGF-1 receptor (IGF1R) in SST-expressing cells (33). Despite the well-known role of SST neurons in regulating GH secretion and acting as a negative feedback node, deleting either GHR or IGF1R had no significant effect on GH secretion (33). However, when both receptors were inactivated in SST neurons, the mice showed increased GH secretion and GH pulse amplitude (33), demonstrating that GHR and IGF1R signaling act redundantly in these cells to control GH secretion.

D2R knockout male mice exhibit reduced body growth and GH secretion, likely due to the absence of central D2R signaling (21–23). In our study, we aimed to replicate this phenotype by deleting D2R in GHRH or SST neurons. It is important to note that D2R in the brain primarily functions as a dopamine autoreceptor (44). As a result, D2R is expressed presynaptically, and its activation inhibits dopamine neuron activity and dopamine release (44). Consequently, most dopamine/TH neurons express D2R to autoregulate their activity. However, D2R can also act postsynaptically, mediating inhibitory effects of dopamine (44). We found that both ARH^GHRH^ and PV^SST^ neurons expressed *Drd2* mRNA. In GHRH neurons that also express TH (approximately 15-20% of GHRH neurons), deleting D2R removed both pre- and post-synaptic effects mediated by this receptor. In GHRH neurons lacking TH (most cells), or in SST neurons, D2R inactivation led to the loss of postsynaptic effects only.

Both GHRH^ΔDrd2^ and SST^ΔDrd2^ male mice showed slight reductions in body weight and lean mass, compared to controls, although this effect reached statistical significance only in SST^ΔDrd2^ mice. Like whole-body D2R knockout mice (21, 22), the reduced body growth was observed only in male mice. Thus, at least in part, we were able to reproduce the D2R knockout phenotype by deleting D2R only in GHRH or SST neurons. The minor reduction in growth observed in GHRH^ΔDrd2^ and SST^ΔDrd2^ male mice likely reflects compensatory or redundant mechanisms. It would be interesting if future studies could generate a mouse with simultaneous D2R ablation in these two neural populations to determine whether D2R expression in one population can compensate for its absence in the other.

Interestingly, GHRH^ΔDrd2^ male mice exhibited reduced basal GH secretion, while total or pulsatile GH secretion remained similar across the experimental groups. Some studies have shown that GH pulses are essential for stimulating growth and IGF-1 secretion (45, 46). Therefore, a decrease in basal GH secretion is unlikely to have a significant effect on body growth or hepatic IGF-1 production (28). This is supported by our findings that show no change in serum IGF-1 levels in mice without alterations in pulsatile GH secretion. The mechanism underlying the alteration in basal secretion in GHRH^ΔDrd2^ mice remains to be further investigated. Additionally, it remains unclear why the effects of D2R ablation on GH secretion and growth are limited to males.

The regulation of GH secretion by dopamine is complex and sometimes appears contradictory. Dopamine stimulates GH secretion in healthy individuals (47), whereas D2R agonists reduce GH secretion in patients with acromegaly (19, 20). Interestingly, dopamine infusion also inhibits GH secretion in the human newborn, whose GH secretion is naturally elevated (48). Dopamine neurons in the hypothalamus express both GHR and IGF1R. While GHR ablation in TH-expressing cells increases GH secretion and body weight (13), IGF1R inactivation in these cells causes a slight reduction in body growth (18), suggesting that GH and IGF-1 act differently in dopamine neurons to control the GH axis. By investigating the consequences of TH ablation in GHRH neurons and of D2R inactivation in either GHRH or SST neurons, our study provides novel and relevant insights into how the dopamine system regulates GH secretion.

In summary, although a subset of ARH^GHRH^ neurons expresses TH, genetic inactivation of this gene is insufficient to cause significant effects on body growth or GH secretion. *Drd2* mRNA expression is observed in ARH^GHRH^ and PV^SST^ neurons. However, D2R removal in these neuronal groups results in minor effects on the GH axis and body growth, limited to male mice, similar to what is observed in D2R knockout mice (21, 22). Therefore, these results suggest that D2R expression in GHRH and SST neurons only plays a small role in the effects caused by the complete loss of D2R in male mice.

## Data Availability

The datasets presented in this study can be found in online repositories. The names of the repository/repositories and accession number(s) can be found below: https://repositorio.uspdigital.usp.br/bitstream/handle/item/827/Reposit%c3%b3rio.xlsx?sequence=-1&isAllowed=y, none.
